# Marked Prolongation of Capillary Refill Time in the Right Foot of a 20-Month-Old Female Infant With Rhabdomyosarcoma

**DOI:** 10.7759/cureus.91142

**Published:** 2025-08-27

**Authors:** Hiroko Fukushima, Sakura Kawahara, Yuni Yamaki

**Affiliations:** 1 Department of Child Health, University of Tsukuba, Tsukuba, JPN; 2 Department of Pediatrics, University of Tsukuba Hospital, Tsukuba, JPN

**Keywords:** capillary refill time, insufficient blood flow, oncologic emergency, pelvic tumor, rhabdomyosarcoma

## Abstract

Childhood cancer is a rare condition. Motor dysfunction may be an initial manifestation of a solid tumor, particularly when caused by spinal or vascular compression. However, in infants who have only recently begun to walk, such symptoms may be misinterpreted as normal developmental variations. Additionally, vascular signs such as delayed capillary refill time (CRT) are extremely rare and may easily be overlooked in clinical practice. Prompt diagnosis and intervention are essential to preserve neurological function in cases of spinal cord compression. Nevertheless, due to the rarity of such presentations, primary care providers, including pediatricians, may have limited experience. A 20-month-old girl presented with right lower limb weakness, bladder and bowel dysfunction, and fever. She had begun walking at 12 months but developed a limp at 18 months. One month prior to admission, she exhibited sensory deficits and diminished reflexes in the right leg, which were initially attributed to her history of prematurity at birth. At 20 months of age, she stopped using her right leg entirely and was admitted to our hospital. Physical examination revealed decreased muscle strength of the right lower limb (knee and ankle joints, manual muscle test grade 4), coldness and prolonged CRT in the right foot, and right thigh swelling (right 26 cm; left 23.5 cm). Computed tomography revealed a large pelvic mass compressing the vasculature and spinal cord, along with another mass from T7 to T9. Multimodal chemotherapy was initiated immediately prior to establishing a final diagnosis. Capillary refill improved within one week, and the thigh asymmetry resolved by day 11. The final histological diagnosis confirmed stage 4 embryonal rhabdomyosarcoma. A very good partial response was achieved after 12 months of treatment. She regained partial ambulation using a brace; however, bladder and bowel dysfunction persisted. This case highlights the diagnostic challenges in identifying malignancy in young children presenting with subtle neurological or vascular symptoms. Previous reports on impaired blood flow in the lower extremities among pediatric cancer patients, including those with rhabdomyosarcoma, have been limited. Although rarely reported in the literature, such presentations are occasionally encountered in daily clinical practice. Previous studies have shown that delayed diagnosis of spinal cord compression is associated with poorer neurological outcomes, particularly in younger patients. Earlier recognition of asymmetry and impaired perfusion in the lower limbs might have enabled more timely diagnosis and intervention in this case.

## Introduction

Childhood cancer is a rare disease, affecting approximately one in every 10,000 children [1]. Patients with solid tumors often present with motor dysfunction, particularly when symptoms are caused by tumor compression. When a child is seen by a physician without specialized training in pediatrics, or even by a pediatrician with limited experience in diagnosing cancer, recognition or consideration of malignancy can be challenging. Moreover, symptoms such as tetraplegia may be difficult to identify in infants who have only recently begun to walk, resulting in delayed diagnosis.

Spinal cord compression occurs in approximately 4% to 5% of pediatric cancer cases [2], and prompt diagnosis and therapeutic intervention are critical for preserving limb function and for the overall prognosis, particularly in emergent situations such as spinal cord emergencies [3]. Unlike adult cancers, childhood cancers tend to progress rapidly and to change in status over a short period, underscoring the importance of early detection.

While the diagnostic skills necessary to evaluate gait disturbance or impaired lower limb perfusion due to childhood cancer are available, the low incidence of such cancers limits clinical exposure in pediatric primary care settings. Consequently, opportunities to develop and refine diagnostic acumen in this context remain limited. Additionally, time to diagnosis may be prolonged due to challenges in conducting imaging studies in infants, including the frequent need for sedation. 

Rhabdomyosarcoma can develop in various anatomical locations, with its clinical presentation and prognosis largely influenced by the tumor site. For instance, orbital tumors commonly lead to proptosis, while those located in the extremities typically present as painless swellings. When arising in the bladder or prostate, the tumor may cause symptoms such as urinary obstruction, visible blood in the urine, or the presence of an abdominal mass. Lesions in deeper regions like the mediastinum or retroperitoneum often grow substantially before becoming clinically apparent [4].

In this report, we describe a case involving a large pelvic tumor that caused both impaired limb perfusion due to vascular compression and spinal cord compression. We hope this case contributes to improving awareness and diagnostic practices in pediatric primary care.

## Case presentation

A 20-month-old girl was admitted to the hospital because of right lower limb weakness, cystorectal dysfunction, and fever.

**Figure 1:**
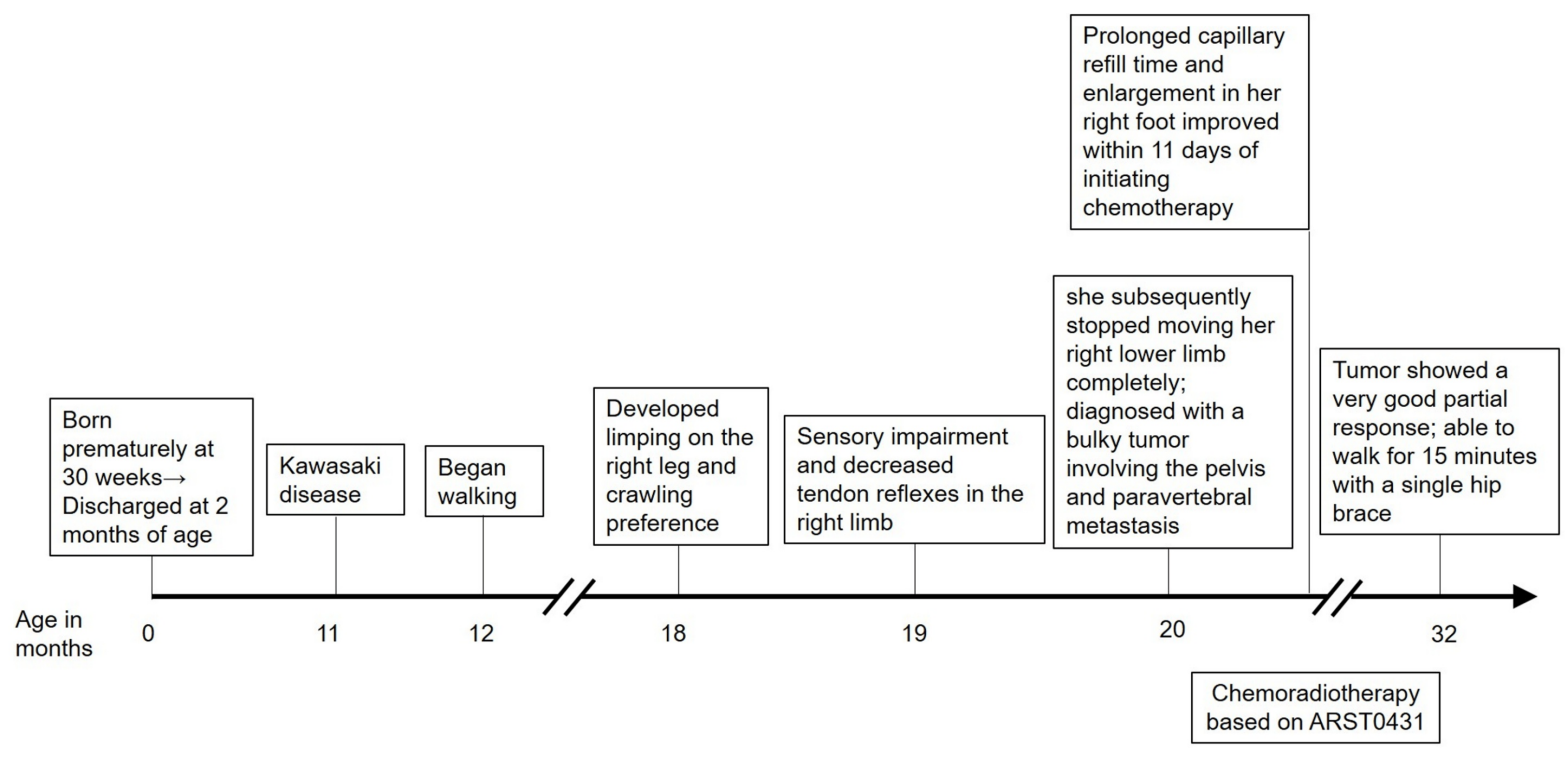
Clinical course of her symptom and treatment Clinical course of the patient showing the progression of lower limb motor impairment, the timing of therapeutic interventions, and the tumor status at the last follow-up.

She was born at 30 weeks and six days’ gestation, with a birth weight of 1023 g. Her hospitalization as a preterm infant passed well, and she was discharged home at two months of age without any medical care. At the age of 11 months, she developed Kawasaki disease, from which she recovered without severe complications. She began walking by herself at 12 months of age, but two months before admission, she started limping on her right leg and crawling to move around. One month before admission, her primary orthopedic surgeon diagnosed sensory impairment in her right lower limb and decreased patellar and Achilles tendon reflexes that were considered due to her being born preterm. She was admitted to our hospital at 20 months of age because she subsequently stopped moving her right lower limb completely (Figure 1).

**Figure 2:**
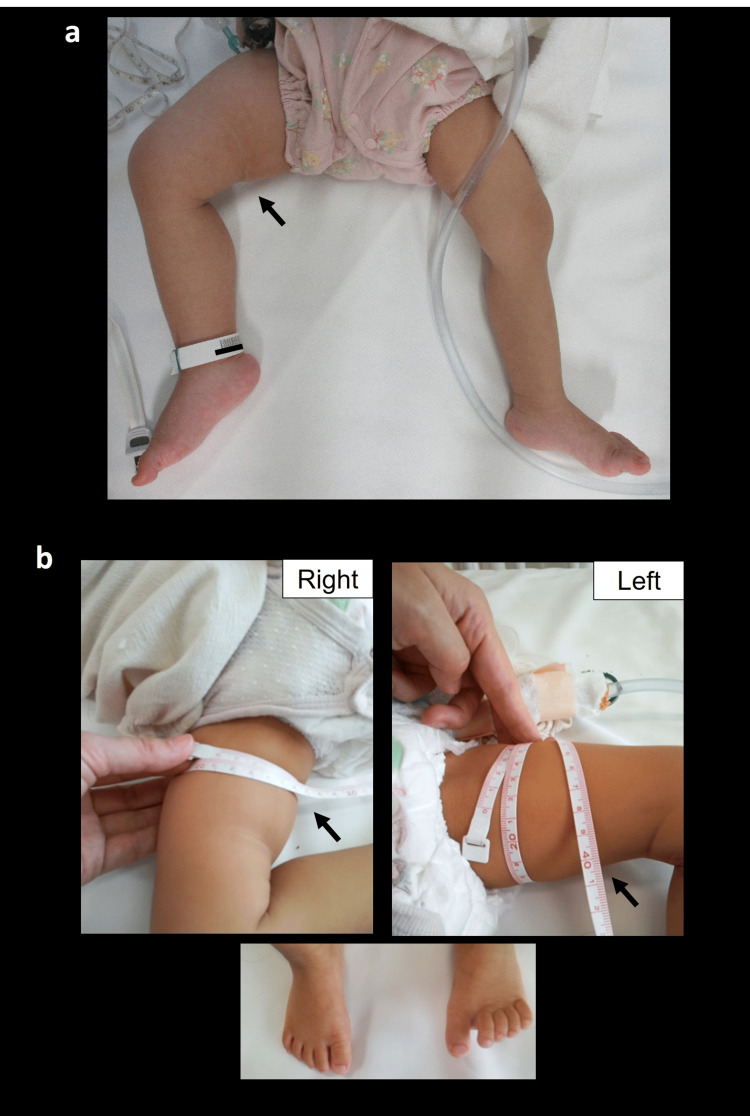
Unilateral thigh enlargement a: Marked enlargement of the right thigh at diagnosis; b: Improvement of the enlargement in the right thigh and foot observed 11 days after initiation of chemotherapy.

On admission, the physical examination revealed decreased right foot muscular strength (manual muscle test 4), decreased right foot temperature, increased right thigh diameter (right 26 cm, left 23.5 cm, Figure 2a), and impaired blood flow with markedly prolonged capillary refill time (CRT) of the right foot (Video 1).

**Video 1:** Prolonged capillary refill time of the right foot Physical examination revealed a markedly prolonged capillary refill time of the right lower foot, but not of the left.

**Figure 3:**
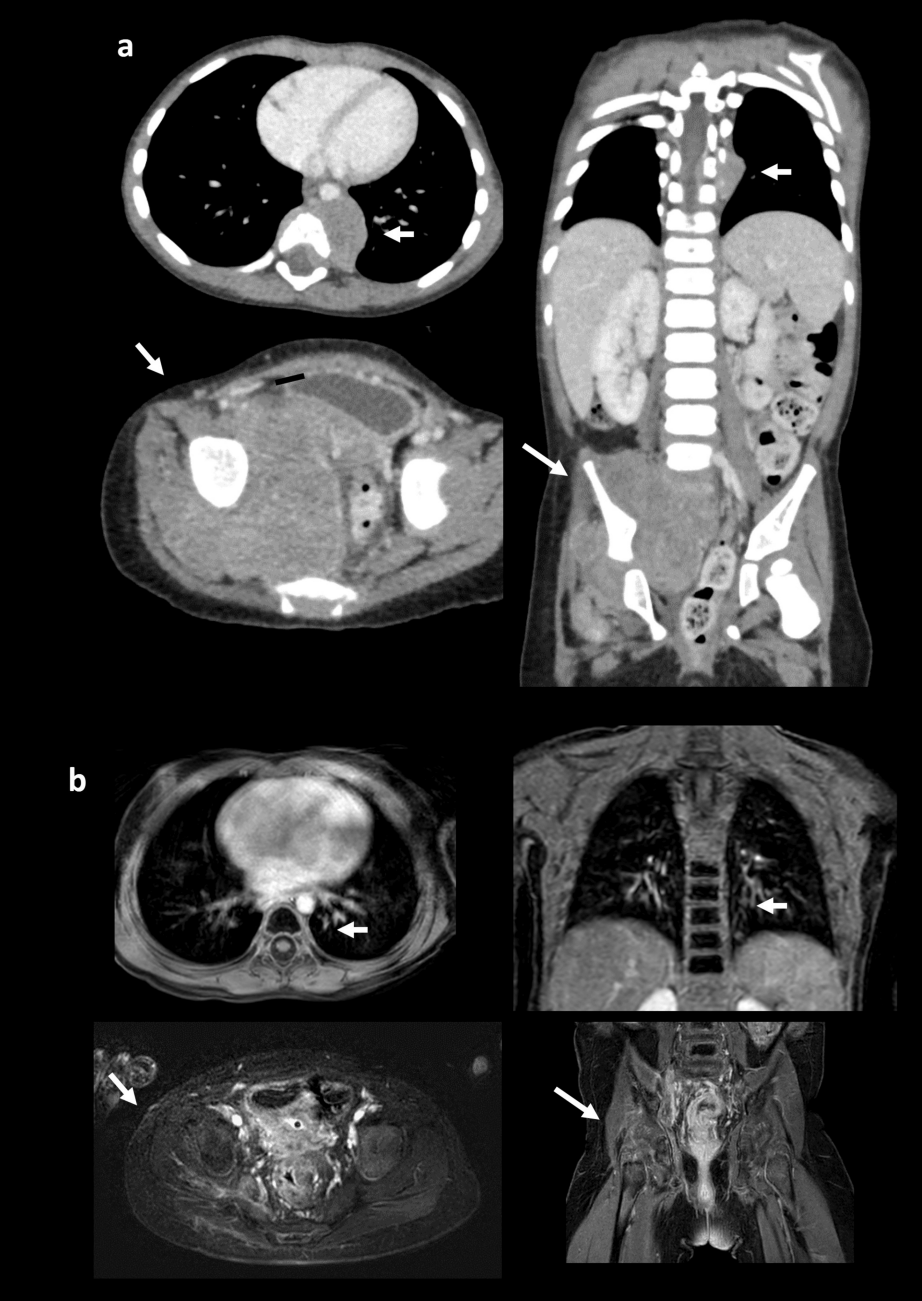
A bulky tumor of the pelvis and parathoracic vertebral metastasis a: Computed tomography revealed a bulky tumor in the right pelvis extending into the pelvic bone and spinal cord, and another tumor located at the seventh to eighth parathoracic vertebrae. b: Magnetic resonance imaging 12 months after diagnosis showed shrinkage of the metastatic tumor in the thoracic spine as well as the primary pelvic tumor.

Computed tomography showed a massive tumor surrounding and extending into the pelvic bone and spinal cord, and another tumor at the seventh to ninth thoracic vertebrae extending into the spinal canal (Figure 3a).

Malignant spinal cord compression was diagnosed, and multiagent chemotherapy of 05A1 consisting of cyclophosphamide 1.2 g/m² on day 1, vincristine 1.5 mg/m² on day 1, pirarubicin 40 mg/m² on day 3, and cisplatin 20 mg/m² on days 1 to 5 was initiated with a provisional diagnosis of neuroblastoma [5]. The CRT improved within a week, and the femoral diameter difference had disappeared 11 days after the start of chemotherapy (Figure 2b). Rhabdomyosarcoma (embryonal type, stage 4) was subsequently diagnosed, and multimodal chemotherapy for rhabdomyosarcoma ARST0431 was started [6, 7]. The tumor shrank, and a very good partial response was achieved at 12 months after diagnosis (Figure 3b). She was able to walk for 15 minutes unaided with a single hip brace, but her bladder and bowel dysfunction persisted (Figure 1).

## Discussion

Blood flow disturbances in the lower extremities have been reported in two pediatric cancer patients with osteochondroma [8]; however, the frequency remains unknown and is presumed to be rare owing to the lack of previous reports. In previous reports, lower limb hypoperfusion in pediatric patients has often been described as acute thrombosis [9,10]. Among children with cancer, acute thrombotic events have been reported more frequently than tumor compression as a cause of reduced blood flow. However, in actual clinical practice, we occasionally encounter cases in which tumors compress surrounding vessels, leading to hypoperfusion or edema distal to the tumor. Since many pediatric cancer patients receive urgent chemotherapy soon after diagnosis, their symptoms tend to improve rapidly, and such findings may therefore be underreported. To our knowledge, this is the first reported case describing lower limb hypoperfusion in a child with rhabdomyosarcoma.

Our patient did not undergo a detailed imaging study or other close examination until two months after the onset of symptoms. In adults, the sudden onset of gait difficulty lasting for two months often leads to significant social challenges and prompts a thorough medical evaluation. In contrast, in infants who have only recently begun to walk, gait disturbances are frequently overlooked. The median time from symptom onset to diagnosis for pediatric cancer patients experiencing their first episode of spinal cord compression due to malignancy was 23 days, with a range of 0 to 360 days, in a previous study [11]. That study also found that younger patients and those with more severe symptoms due to spinal cord compression at diagnosis were associated with worse long-term neurological complications [8]. Our patient may have exhibited mild symptoms of spinal cord compression, which could have enabled earlier diagnosis and therapeutic intervention if health care professionals had considered malignancy or other serious but reversible conditions and had more carefully examined left-right asymmetries and decreased lower extremity perfusion, even in this young child. Our patient presented with key clinical features suggestive of both limb ischemia and spinal cord compression (Table 1) [10, 12]. 

**Table 1:** Symptoms of limb ischemia and spinal cord compression Sources: [10,12]

Limb ischemia	Spinal cord compression
Paresthesia	Motor deficit
Pain	Pain
Pallor	Irritability
Pulselessness	Sphincter dysfunction
Muscle weakness	Respiratory distress
Cool, pale and mottled skin	

In pediatric patients with limb muscle weakness, routine evaluation of CRT and limb circumference asymmetry may aid in the earlier detection of underlying pathological conditions, including those associated with tumor mass effects.

Another study showed the diagnostic delay of children with cancer may be longer for the physician with whom the patient contacted first [13]. Based on these previous reports, it was considered that the time interval and process from the onset of symptoms in pediatric patients to the suspicion of childhood cancer, subsequent further investigation, and establishment of the final diagnosis may be influenced by the diagnostic skills, experience, and knowledge of the physician who initially evaluated the patient. Nevertheless, early diagnosis of childhood cancer can be achieved through a careful whole-body physical examination and thoughtful consideration of potential underlying causes, supported by knowledge of previously reported cases, even in the absence of personal experience.

## Conclusions

We treated a child who had decreased blood flow and swelling in the lower extremity and gait disturbance. Although this patient exhibited abnormal limb movement from an early stage, neither CRT nor limb circumference asymmetry was assessed. Had these evaluations been performed earlier, the presence of the tumor might have been detected at an earlier point. Careful and thorough examination may facilitate earlier diagnoses of children with cancer, thereby contributing to reductions in cancer-related mortality and long-term physical impairment.
